# Antibiotic resistance associated lactic acid cross tolerance in Shiga-toxin producing *E. coli*

**DOI:** 10.3389/fmicb.2023.1059144

**Published:** 2023-04-26

**Authors:** Ikechukwu Oguadinma, Abhinav Mishra, Govindaraj Dev Kumar

**Affiliations:** ^1^Center for Food Safety, The University of Georgia, Griffin, GA, United States; ^2^Department of Food Science & Technology, The University of Georgia, Athens, GA, United States

**Keywords:** antibiotic resistance, Shiga-toxin producing *E. coli*, lactic acid, MIC, cross-tolerance

## Abstract

**Introduction:**

The occurrence of antibiotic resistant (ABR) bacteria in foods is a growing public health challenge. We evaluated sanitizer cross-tolerance among ABR *Escherichia coli* (*E. coli*) O157:H7 and non-O157:H7 Shiga-toxin producing *E. coli* (STEC) serogroups. Sanitizer tolerance in STEC could be a public health concern as mitigation strategies against the pathogen might be compromised.

**Methods:**

Resistance to ampicillin and streptomycin were evolved in *E. coli* serogroups: O157:H7 (H1730, and ATCC 43895), O121:H19 and O26:H11. Resistance to antibiotics was evolved chromosomally through incremental exposure to ampicillin (amp C) and streptomycin (strep C). Transformation using a plasmid was performed to confer resistance to ampicillin to generate amp P strep C.

**Results:**

The minimum inhibitory concentration (MIC) of lactic acid for all strains evaluated was 0.375% v/v. Analysis of bacterial growth parameters in tryptic soy broth amended with 0.0625% v/v, 0.125% v/v, and 0.25% v/v (subMIC) lactic acid indicated that growth correlated positively with the lag phase duration, and negatively with both the maximum growth rate and change in population density for all strains evaluated except for the highly tolerant variant- O157:H7 amp P strep C. Strains O121 NR (non-ABR), O121 amp C, O121 amp P strep C, O157:H7 H1730 amp C and O157:H7 H1730 amp P strep C were not inactivated after exposure to 1% and 2.5% v/v lactic acid for 300 s. No recovery of cells was observed after the strains were exposed to 5% v/v lactic acid for 300 s. ABR strains O157:H7 H1730 amp C and O157: H7 H1730 amp P strep C demonstrated a high tolerance to lactic acid (*P* ≤ 0.05).

**Conclusion:**

ABR in isolate *E. coli* O157: H7 H1730 may improve tolerance to lactic acid. Increased tolerance may be discerned by evaluating growth parameters of bacteria in presence of sub-MIC levels of lactic acid.

## Highlights

- The MIC of lactic acid was the same for both O157 and non-O157 STEC.
- Increasing lactic acid concentrations at sub-MIC correlated positively with the Lag phase duration (L) and negatively for maximum growth rate (μmax) and change in population density (A).
- *E. coli* O157:H7 H1730 amp C and *E. coli* O157:H7 H1730 amp P strep C were the most tolerant to lactic acid.

## Introduction

The contamination of foods by Shiga-toxin producing *Escherichia coli* (*E. coli*) (STEC) is a recurring issue that significantly impacts public health. *E. coli* O157:H7 is the most commonly implicated STEC serogroup and is responsible for 40.3% of domestically acquired illnesses in the United States (US) (Hale et al., 2012). Emerging pathogens of concern such as STEC serogroups O26, O45, O103, O111, O121, and O145 are highly prevalent in cattle rearing environments (Brooks et al., 2005) and have been added to the list of adulterants in beef (Wheeler et al., 2014).

Illnesses resulting from STEC infection could range from a mild, self-limiting gastroenteritis to the development of hemorrhagic colitis (bloody diarrhea) in 90% of patients and hemolytic uremic syndrome (HUS) in 5–15% of patients (Smith et al., 2014). HUS is typically characterized by acute kidney failure, microangiopathic hemolytic anemia (damage of small blood vessels with destruction of red blood cells), and thrombocytopenia (decrease in platelets) (Smith et al., 2014). Despite the severity of STEC infections, antibiotic use is contraindicated, since exposure to certain antibiotics could induce Shiga toxin (Stx) production (Zhang et al., 2000). Resistance to antibiotics in STEC can occur during cattle production (Centers for Disease Control and Prevention, 2020). Antibiotics are commonly administered to cattle to treat various infections and improve feed efficiency (McEwen and Fedorka-Cray, 2002). Long-term exposure to antibiotics and other antimicrobials at sub-lethal concentrations can induce the development of resistance in bacteria (Oniciuc et al., 2019). Furthermore, resistance to one antimicrobial group can result in cross-tolerance to other antimicrobials (Gnanadhas et al., 2013). Products such as beef cuts and ground beef have been routinely recalled due to the presence of STEC (Centers for Disease Control and Prevention, 2022) despite sanitizer use on carcasses (Winkler and Harris, 2009). Lactic acid is a commonly used sanitizer during beef processing (Castillo et al., 2001; Winkler and Harris, 2009; Beier et al., 2013). Lactic acid disrupts transmembrane proton motive force and reduces intracellular pH of bacteria by penetrating the cytoplasmic membrane of cells in its undissociated form (Alakomi et al., 2000). It is used as a spray or wash on beef carcasses at concentrations ranging from 2 to 5% (Winkler and Harris, 2009). Use of lactic acid has also been explored as an intervention during the tempering of wheat as a strategy to reduce the prevalence of STEC in flour (Sabillón et al., 2016).

The continued foodborne outbreaks associated with *E. coli* O157:H7 in beef despite consistent use of sanitizers could be occurring because of the pathogens ability to adapt to acid stress through acid resistance systems (AR) (Foster, 2004; Lu et al., 2013) and efflux pumps (Deininger et al., 2011). Previous research has indicated that tolerance of an antibiotic resistant (ABR) lettuce isolate, *E. coli* O157:H7 H1730 to lactic acid was linked to increased efflux pump activity in ABR *E. coli* O157:H7 (Oguadinma et al., 2022a). Further, it has been observed that certain types of antibiotic resistance confer higher tolerance to stressors and sanitizers than others (Hayman et al., 2022; Oguadinma et al., 2022a,b). It is not known if the occurrence of similar patterns of antibiotic resistance in other isolates of *E. coli* O157:H7 and STEC serogroups would result in a similar increase in tolerance to lactic acid.

The objectives of this study were to evaluate if antibiotic resistance in outbreak associated isolates of *E. coli* O157:H7, *E. coli* O26:H11, *E. coli* O121:H19 would result in cross-tolerance to commercially used concentrations of lactic acid. Growth rate parameters of non-resistant parent strains and ABR variants were evaluated as predictors of elevated antibiotic tolerance.

## Materials and methods

### Bacterial strains

**FIGURE 1:**
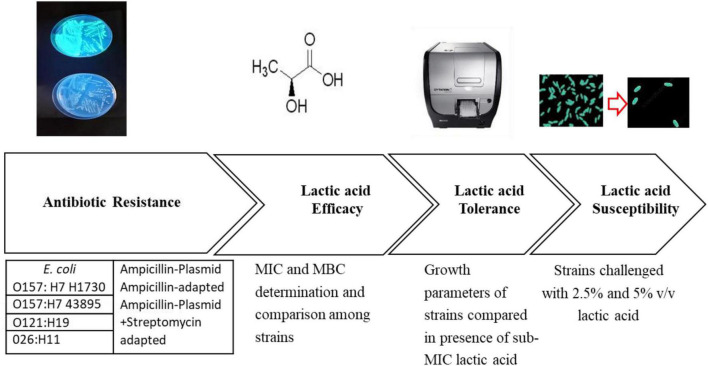
Experimental overview.

Three serogroups of Shiga toxin-producing *E. coli* were used in this study. *E. coli* O157:H7 H1730 was a human isolate from a lettuce outbreak and *E. coli* O157:H7 (ATCC 43895) was from a 1982 ground beef outbreak. Both isolates were obtained from the culture collection at the Center for Food Safety, University of Georgia. *E. coli* O121:H19 (strain TW08980) and *E. coli* O26:H11 (strain 3012-03) were from a 2016 Missouri flour outbreak and obtained from the Michigan State University STEC Center. Isolates were revived from frozen storage by transferring to Tryptic Soy Broth (TSB, Neogen, Lansing, MI, USA) and incubating at 37°C for 24 h. The bacterial strains were evaluated for antibiotic resistance to ampicillin and streptomycin by streaking on Tryptic Soy Agar (TSA; Neogen Lansing, MI, USA) amended with 100 μg/ml of ampicillin, 100 μg/ml of streptomycin, and a combination of 100 μg/ml streptomycin and ampicillin. Plates were placed at an incubation temperature of 37°C for 24 h and observed for colony formation. Cultures that did not yield colonies in the antibiotic amended plates were considered to be susceptible to the antibiotics (Table 1 and Figure 1).

**TABLE 1:** List of Shiga toxin producing *E. coli* strain variants used in this study.

Bacterial strain	Mode of resistance	Strain name for this study
*Escherichia coli* O157:H7 H1730	No resistance (parent strain)	O157:H7 H1730 NR
*E. coli* O157:H7 H1730 amp resistant	Chromosome	O157:H7 H1730 amp C
*E. coli* O157:H7 H1730 amp and strep resistant	Plasmid and chromosome	O157:H7 H1730 amp P strep C
*E. coli* O157:H7 43895	No resistance (Parent strain)	O157:H7 43895 NR
*E. coli* O157:H7 43895 amp resistant	Chromosome	O157:H7 43895-amp C
*E. coli* O157:H7 43895 amp and strep resistant	Plasmid and chromosome	O157:H7 43895-amp P strep C
*E. coli* O26:H11	No resistance (parent strain)	O26:H11 NR
*E. coli* O26:H11 amp resistant	Chromosome	O26:H11 amp C
*E. coli* O26:H11 amp and strep resistant	Plasmid and chromosome	O26:H11 amp P strep C
*E. coli* O121:H19	No resistance (parent strain)	O121:H19 NR
*E. coli* O121:H19 amp resistant	Chromosome	O121:H19 amp C
*E. coli* O121:H19 amp and strep resistant	Plasmid and chromosome	O121:H19 amp P strep C

### Ampicillin and streptomycin adaptation among STEC

#### Chromosomal resistance

Chromosomal resistance to streptomycin and ampicillin was evolved by sequentially transferring the bacterial strains into increasing concentrations of the antibiotics. Briefly, 100 μl of culture from a lower antibiotic concentration was transferred to fresh media containing 900 μl a higher antibiotic concentration. This process was repeated with a 10 μg/ml increase in the antibiotic concentration for each transfer until adaptation to 100 μg/ml of antibiotic was achieved (Table 1 and Figure 1).

#### Transformation by electroporation

Transformation of cells to take up a green fluorescence ampicillin resistance plasmid (GFP amp plasmid) was performed following the method described by Kumar et al. (2017) with minor modifications. Briefly, competent cells were prepared as follows: 45 ml of Tryptic soy broth (TSB) was inoculated with 1 ml of overnight cultures of the bacterial strains. Cultures were incubated at 37 °C for 4 h to achieve an optical density (OD_600 *nm*_) of 0.8 and then placed in ice for 15 min. The cultures were then centrifuged at 1400 × *g* for 10 min to pellet the cells, and the supernatant discarded. The pelleted cells were washed three times with 15% ice-cold glycerol and stored at −20°C until use. The cells were transformed using the host range plasmid pGFPuv (SnapGene, 2021). Electroporation conditions applied were 2.5 kV, 25 μF, and 400 Ω using the Gene Pulser II system (Bio-Rad, Hercules CA, USA). Colonies of transformed cells expressed fluorescence upon excitation with UV light (365 nm) and were resistant to 100 μg/ml ampicillin (Table 1 and Figure 1).

### Preparation of bacterial inoculum

Stock cultures of the bacterial strains were prepared by streaking each strain to TSA or TSA + 100 μg/ml of antibiotics (TSA + Amp, TSA + Amp + Strep) and incubating at 37°C for 24 h. Colonies from overnight cultures were scraped from the plates with a sterile loop and suspended in phosphate-buffered saline (1 × PBS; VWR International, Radnor, PA, USA). The bacterial population was adjusted to 6.42 ± 0.11 log CFU/ml.

### Determination of the minimum inhibitory concentration (MIC) of lactic acid

The minimum inhibitory concentration of lactic acid (LA, L-lactic acid, Xena International Inc., IL, USA) for the antibiotic-resistant and non-resistant bacterial strains was determined using a 96 well plate broth dilution method described by Dev Kumar et al. (2020) with some modifications. Briefly, lactic acid stock solutions were serially diluted in 96 well plates −180 μl in each well—and inoculated with 20 μl of 6.83 ± 0.18 log CFU/ml of bacteria. Serial dilutions were performed from initial lactic acid concentrations of 5 and 3% to obtain lactic acid concentrations of 2.5, 1.5, 1.25, 0.75, 0.62, 0.37%, 0.31%, 0.18, 0.15, 0.09, 0.07, 0.04, 0.03, and 0.02% v/v. The 96-well plates (Costar^®^ 96 Well Flat Bottom, Corning LifeSciences Inc. ME, USA) were incubated for 24 h at 37 °C, and the growth kinetics were observed using the Bio-Tek Cytation 3 multi-mode plate reader (BioTek Instruments, Inc. USA). Conditions in the Bio-Tek Cytation 3 multi-mode plate reader were set as follows: the total runtime was set at 24 h with read intervals of 30 min, the shaker was set to an orbital shake every 10 s at a frequency of 283 cpm (3 mm), the read speed was set to Normal with a delay of 100 ms and the optical density was read at an absorbance of 600 nm. Un-inoculated blanks of TSB were used as a control for this experiment (Figure 1).

### Bactericidal concentration of lactic acid

A commercial food grade lactic acid (88% v/v) was obtained from Xena Inc (Xena International Inc; Polo IL, USA) and lactic acid solutions were prepared in TSB at concentrations of 0.5, 1, 1.5, 2, 2.5, and 5% v/v. The pH of lactic acid was determined using a pH meter (Oakton pH 510 Benchtop Meter, Oakton Instruments, Vernon Hills, IL, USA) with a sensitivity of 0.01 and two-point calibration. The concentration at which lactic acid could prevent survival and regrowth of the STEC strains was considered as the bactericidal concentration. The bactericidal concentration of lactic acid for all the bacterial strains was evaluated as follows: Each 900 μl of 0.5, 1, 1.5, 2, 2.5, and 5% v/v lactic acid was inoculated with 100 μl of the bacteria for 300 s. Solution was centrifuged immediately after exposure for 1 min at 13000 × *g* using a Corning high speed microcentrifuge (Corning Inc., Corning, NY, USA). The supernatant was discarded, and pellets were resuspended in 1 ml sterile deionized water (SDW). From the resuspended solution, 100 μl was transferred to 100 μl of 2 × TSB in 96-well plates (Costar^®^ 96 Well Flat Bottom, Corning Life Sciences Inc. ME, USA) and incubated for 24 h at 37 °C. The plates were observed for turbidity to determine survival and regrowth after incubation by determining the OD_600 *nm*_ using the BioTek Cytation multi-mode plate reader (Figure 1).

### Evaluation of bacterial growth rates at sub-lethal concentrations of lactic acid

The growth rates for the different bacterial strains in TSB and the subminimum concentration of lactic acid were evaluated using the turbidimetric technique. The experiment was conducted in a 96 well microplate by inoculating 20 μl of 6.76 ± 0.71 log CFU/ml of bacteria to 180 μl of media. Growth rates were observed for 24 h at 37 °C using the Bio-Tek Cytation 3 image reader (BioTek Instruments, Inc. USA). Conditions in the Bio-Tek Cytation 3 image reader were set as described previously. Three biological and three technical replicates were performed, and strains exposed to TSB without the antimicrobial stress were used as controls.

### Mathematical modeling for bacterial growth

The modified Gompertz model (Gibson et al., 1988) modified by (Begot et al., 1996) was fitted to the growth curve of these bacterial strains using MATLAB software (version R2021a, The MathWorks, Inc. Natick, MA, USA). The model can be described by the following equation (Hayman et al., 2022), where *N* is the bacterial population at a given time, N_0_ is the initial bacterial population, O.D_*min*_ is the lowest O.D. value above the detection threshold, *A* is the logarithmic increase of bacterial population, *L* is the lag time, μ is the maximum growth rate, and *t* is time:


$$l\,o\,g_{10}\,\left(\frac{N}{N_{0}}\right)=l\,o\,g_{10}\,\left(\frac{{\left(△O.D.\right)}_{t}}{△O.D._{m\,i\,n}}\right)$$

$$=A\cdot e\,x\,p\,\left(-e\,x\,p\,\left(\frac{\mu \cdot e}{A}\cdot \left(L-t\right)+1\right)\right)$$

The growth parameters assessed were change in bacterial population in log CFU/ml (*A*), lag phase duration in hours (*L*), and maximum growth rate in log CFU/h (μ _*max*_).

### Bactericidal concentration of lactic acid

A commercial food grade lactic acid (88% v/v) was obtained from Xena Inc (Xena International Inc; Polo IL, USA) and lactic acid solutions were prepared in TSB at concentrations of 0.5, 1, 1.5, 2, 2.5, and 5% v/v. The pH of lactic acid was determined using a pH meter (Oakton pH 510 Benchtop Meter, Oakton Instruments, Vernon Hills, IL, USA) with a sensitivity of 0.01 and 2-point calibration. The concentration at which lactic acid could prevent survival and regrowth of the STEC strains was considered as the bactericidal concentration. The bactericidal concentration of lactic acid for all the bacterial strains was evaluated as follows: Each 900 μl of 0.5, 1, 1.5, 2, 2.5, and 5% v/v lactic acid was inoculated with 100 μl of bacterial culture (6.89 ± 0.72 log CFU/ml) for 300 s. Solution was centrifuged immediately after exposure for 1 min at 13000 × *g* using a Corning high speed microcentrifuge (Corning Inc., Corning, NY, USA). The supernatant was discarded, and pellets were resuspended in 1 ml sterile deionized water (SDW). From the resuspended solution, 100 μl was transferred to 100 μl of 2 × TSB in 96-well plates (Costar^®^ 96 Well Flat Bottom, Corning Life Sciences Inc. ME, USA) and incubated for 24 h at 37°C. The plates were observed for turbidity to determine survival and regrowth after incubation by determining the OD_600 nm_ using the BioTek Cytation 3 multi-mode plate reader.

### Efficacy of 2.5 and 5% v/v lactic acid against STEC serogroups and their ABR variants

Overnight cultures of the bacterial strains grown either on TSA or TSA + 100 μg/ml of ampicillin, and TSA + 100 μg/ml of streptomycin and ampicillin were used to prepare 8 log CFU/ml of bacterial inoculum in 1 × PBS. From the suspended culture, 100 μl was transferred to 900 μl of TSB containing 2.5 or 5% lactic acid for 30 and 300 s. The solution was centrifuged immediately after exposure for 1 min at 13000 × *g* using the Corning LSE high speed microcentrifuge. The supernatant was discarded, and the pellets were resuspended in 1 ml 1 × PBS. Enumeration of bacteria was done using the droplet plate method where colony forming units (CFU) of cells within the perimeter of drop were used for enumerating bacterial populations from serially diluted samples. The number of colonies formed were counted after incubation for 24 h at 37°C. The limit of detection for the assay was 10 cells or 1.00 log CFU/ml.

### Statistical analysis

All experiments were conducted in three biological and three technical replicates. Differences between the bactericidal concentration of lactic acid and decline in population of all bacterial strains evaluated after acid exposure were compared using the one-way analysis of variance (ANOVA). Significant differences between the means were compared using the Tukey’s Honestly Significant Difference (HSD) test at a 0.05 significance level, using JMP statistical software (SAS Institute Inc, USA).

## Results

### Inhibitory concentration of lactic acid against STEC serogroups and their ABR variants

Growth of all STEC strains and their ABR variants (*n* = 12) was inhibited by an MIC 0.375% v/v lactic acid.

**FIGURE 2:**
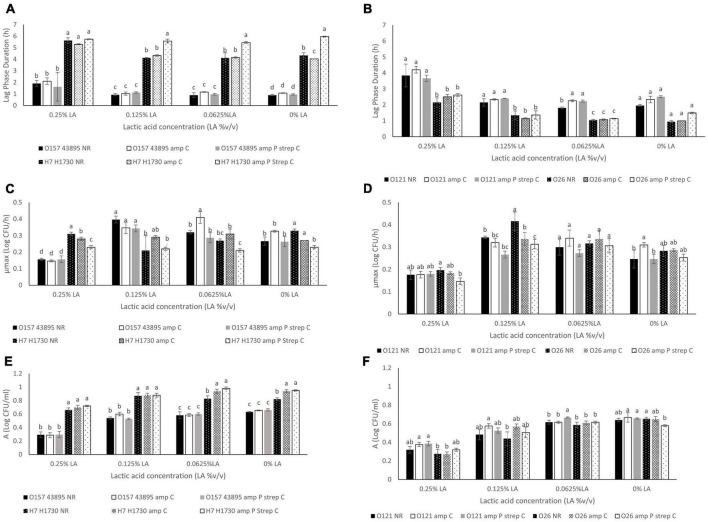
Comparison of **(A)** Lag phase duration (L) for O157:H7 STEC- O157 43895 and O157 H1730, **(B)**. Lag phase duration (L) for non O157:H7 STEC- O121 and O26, **(C)**. Maximum growth rate (μmax) for O157:H7 STEC- O157 43895 and O157 H1730, **(D)**. Maximum growth rate (μmax) for non O157:H7 STEC- O121 and O26, **(E)**. Change in bacterial population (A) for O157:H7 STEC- O157 43895 and O157 H1730 **(F)**. Change in bacterial population (A) for non O157:H7 STEC- O121 and O26. Strains were labeled as NR (no resistance), amp C (chromosomal based resistance to ampicillin) and amp P strep C (plasmid mediated ampicillin resistance and chromosomal streptomycin resistance). Significant differences among strains have been denoted by different alphabet.

**FIGURE 3:**
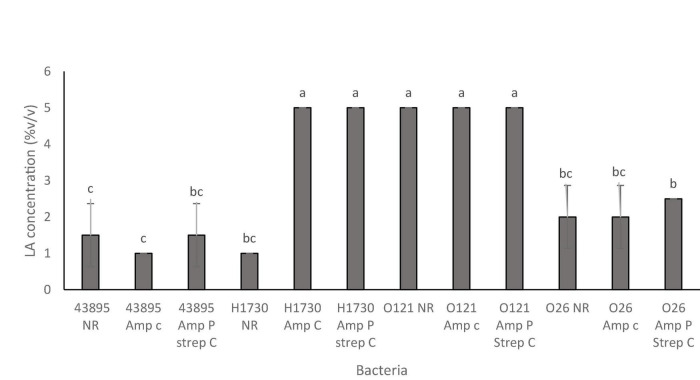
Bactericidal concentrations of lactic acid for the O157:H7 STEC–*E. coli* O157:H7 43895 (43895) and *E. coli* O157:H7 H1730 (H1730) and non O157:H7 STEC- *E. coli* O121:H19 (O121) and *E. coli* O26:H11 (O26) with no resistance (NR), plasmid-based ampicillin resistance (amp P) and a combination of plasmid-based ampicillin resistance and streptomycin resistance through incremental exposure (amp P Strep C). Significant differences among strains have been denoted by different alphabet.

The STEC strains and their ABR variants were grouped as “tolerant,” “moderately susceptible,” and “highly susceptible,” according to the concentration of lactic acid that was required to be bactericidal (Table 2). Tolerant strains in this study were only inhibited by 5% v/v lactic acid, “moderately susceptible” strains were inhibited by 2–2.5% v/v lactic acid and “highly susceptible” strains by 1–1.5% v/v lactic acid (Table 2). Antibiotic resistance did not affect susceptibility of serogroups O157:H7 43895, O26:H11, and O121:H19 to lactic acid (Figure 2). Serogroup O157:H7 43895 and its ABR variants were highly susceptible to lactic acid (Figure 2), serogroup O26:H11 and its ABR variants remained moderately susceptible whereas serogroup O121:H19 and its ABR variants remained tolerant lactic acid (Figure 2). Antibiotic resistance in serogroup O157:H7 H1730 induced “tolerance” to lactic acid while O157:H7 H1730 NR was highly susceptible to lactic (*P* ≤ 0.05) (Figure 3).

**TABLE 2:** Classification of the bacterial strains according to bactericidal concentrations of lactic acid.

Bacteria	Classification	Bactericidal concentration (% v/v)
**Tolerant (5% v/v)**		
O121:H19 NR		5.00 ± 0.00
O121:H19 Amp c		5.00 ± 0.00
O121:H19 Amp P Strep C		5.00 ± 0.00
O157:H7 H1730 Amp C		5.00 ± 0.00
O157:H7 H1730 Amp P strep C		5.00 ± 0.00
**Moderately susceptible (2–2.5% v/v)**		
O26:H11 Amp P Strep C		2.50 ± 0.00
O26:H11 Amp c		2.00 ± 0.86
O26:H11 NR		2.00 ± 0.86
**Highly susceptible (1–1.5% v/v)**		
O157:H7 43895 Amp P strep C		1.50 ± 0.86
O157:H7 43895 NR		1.50 ± 0.86
O157:H7 43895 Amp c		1.00 ± 0.00
O157:H7 H1730 NR		1.00 ± 0.00

### Growth parameters of bacterial strains at sublethal concentrations of lactic acid

Differences in antibiotic resistance (ABR) profile did not affect the MIC of lactic acid which was determined to be 0.375% v/v for all bacterial strains evaluated (*P* > 0.05). Growth parameters of O157:H7 STEC (Figures 2A, C, E) and O121:H19 and O26:H11 (Figures 2B, D, F) at 0.0625, 0.125, and 0.25% v/v lactic acid were evaluated to describe differences between the bacterial strains due to the acquisition of ABR. As observed, the type of strain and concentration of lactic acid affected the growth parameters (*P* ≤ 0.05). Generally, lactic acid concentration of 0.25% v/v significantly increased the average lag phase duration (L) by 1.15 h, decreased the average maximum growth rate (μmax) by 0.08 log CFU/h, and decreased the average change in bacterial population (A) by 0.3 log CFU/ml (*P* ≤ 0.05).

The L, μmax and A for bacterial strain O157:H7 43895 was not significantly different for both the parent strain and the ABR variants across all 3 sublethal lactic acid concentration (*P* > 0.05) except at the μmax for O157:H7 43895 amp C in 0.0625% v/v lactic acid which was significantly different from the other variants (*P* ≤ 0.05) (Figures 2A, E). The μmax for O157:H7 43895 amp C at 0.0625% v/v lactic acid was 0.41 ± 0.03 log CFU/h compared to 0.32 ± 0.01 log CFU/h for O157:H7 43895 NR and 0.28 ± 0.03 log CFU/h for O157:H7 43895 amp P strep C. Growth parameters in the no-lactic acid control was not significantly different for all the strains (*P* > 0.05).

The same pattern was observed for strain O121:H19 at sublethal concentrations of lactic acid. However, significant differences were observed at the L and A of cells exposed to 0.0625% v/v lactic acid. The longest L of 1.81 ± 0.05 h at 0.0625% v/v lactic acid was observed for O121 NR (*P* ≤ 0.05) and the highest A of 0.66 ± 0.01 log CFU/ml was observed for O121 amp P strep C (*P* ≤ 0.05) (Figure 2F). In the controls, the shortest L of 1.96 ± 0.06 h was observed for O121 NR and the highest μmax of 0.31 ± 0.01 log CFU/h was observed for O121 amp C (*P* ≤ 0.05) (Figures 2B, F).

The difference between variants of strain O26:H11 was observed at 0.25% v/v lactic acid. The μmax of 0.14 ± 0.01 log CFU/h for O26 amp P strep C observed at 0.25% v/v lactic acid was significantly different from the μmax of 0.19 ± 0.01 log CFU/h observed for O26 NR (*P* < 0.05) (Figure 2D). In the control, O26 NR had the shortest L of 0.95 ± 0.06 h (*P* ≤ 0.05). The A of 0.65 ± 0.01 observed for O26 NR in the control was significantly different from the A of 0.58 ± 0.01 observed for O26 amp P strep C (*P* ≤ 0.05).

Bacterial strain O157:H7 H1730 amp P strep C had the longest L of 5.97 ± 0.04 h, 5.45 ± 0.08 h, 5.58 ± 0.17 h and 5.74 ± 0.03 h in the absence of lactic acid, 0.0625% v/v lactic acid, 0.125% lactic acid and 0.25% lactic acid, respectively, compared to all other strain variants evaluated (*P* ≤ 0.05) (Figure 2A). At 0.0625% v/v lactic acid, the L of 5.45 ± 0.08 h for O157:H7 H1730 amp P strep C was significantly different from L for O157:H7 H1730 amp C and O157:H7 H1730 NR (*P* ≤ 0.05). The μmax of 0.21 ± 0.01 log CFU/h for O157:H7 H1730 amp P strep C was the smallest at this concentration (*P* ≤ 0.05) but O157:H1730 NR had the smallest A of 0.83 ± 0.04 log CFU/ml compared to the other O157:H7 H1730 strain variants (*P* ≤ 0.05). O157:H7 H1730 amp P strep C had the longest L of 5.58 ± 0.17 h at 0.125% v/v lactic acid and the smallest μmax of 0.23 ± 0.01 log CFU/h at 0.25% v/v lactic acid compared to the other O157:H7 H1730 strain variants (*P* ≤ 0.05).

Increase in the lactic acid concentration of the medium correlated positively with the (L) and negatively with the (μmax) and (A) for all bacterial strains except O157:H7 H1730 amp P strep C that showed a negative correlation with L (*r* = −0.18) and positive correlation with μmax (*r* = 0.21) (Table 3).

**TABLE 3:** Correlation between lactic acid concentration and growth parameters of STEC.

Bacteria	L (h)	μ max (log CFU/h)	A (log CFU/ml)
O157:H7 43895 NR	0.84	-0.48	-0.94
O157:H7 43895 amp C	0.82	-0.78	-0.92
O157:H7 43895 ampP strepC	0.45	-0.56	-0.97
O157:H7 H1730 NR	0.76	-0.07	-0.72
O157:H7 H1730 amp C	0.95	-0.05	-0.93
O157:H7 H1730 amp P strep C	-0.18	0.21	-0.93
O121:H19 NR	0.85	-0.43	-0.96
O121:H19 amp C	0.87	-0.81	-0.95
O121:H19 ampP strepC	0.82	-0.71	-0.95
O26:H11 NR	0.92	-0.39	-0.96
O26:H11 amp C	0.91	-0.66	0.94
O26:H11 amp P strep C	0.8	-0.65	-0.91

### Reduction in the population of bacterial strains exposed to lactic acid

**FIGURE 4:**
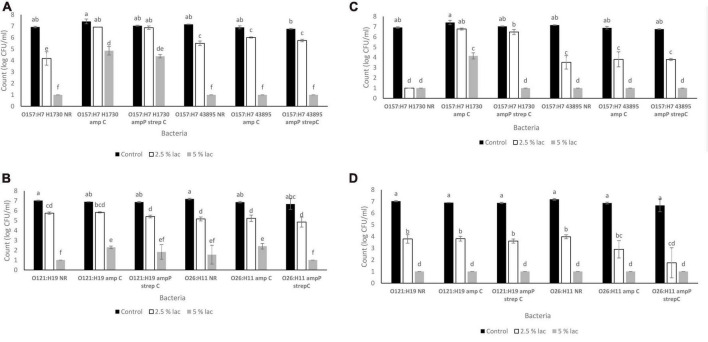
Survival of STEC exposed to lactic acid **(A)**. O157 STEC exposed to lactic acid for 30 s **(B)** O121 and O26 STEC exposed to lactic acid for 30 s **(C)**. O157 STEC exposed to lactic acid for 300 s **(D)**. O121 and O26 STEC exposed to lactic acid for 300 s. Significant differences among strains have been denoted by different alphabet.

The pH of media amended with lactic acid were 2.76 ± 0.00 for 2.5% v/v and 2.5 ± 0.02 for 5% v/v. Survival of the bacterial strains in lactic acid were significantly impacted by both lactic acid concentration (2.5 and 5%) and exposure time (30 and 300 s) (*P* ≤ 0.05) (Figures 4A–D). The highest population decline of 2.74 ± 0.61 log CFU/ml was observed in O157:H7 H1730 NR and the least population decline of 0.17 ± 0.16 was observed in O157:H7 H1730 amp P strep C post exposure to 2.5% v/v lactic acid for 30 s (Figure 4A). After exposure to 2.5% v/v lactic acid for 300 s, O157:H7 H1730 NR and O26 amp P strep C showed the highest population declines of 5.92 ± 0.07 (Figure 4C) and 4.92 ± 1.05 log CFU/ml (Figure 4D) respectively (*P* ≤ 0.05). The most tolerant strains at 2.5% v/v lactic acid for 300 s were O157:H7 H1730 amp C 0.63 ± 0.17 log CFU/ml and O157 H7 H1730 amp P strep C 0.55 ± 0.29 log CFU/ml. The highest tolerance in 5% v/v lactic acid after 30 s exposure was observed in O157:H7 H1730 amp P strep C and O157:H7 H1730 amp C with 2.66 ± 0.14 and 2.56 ± 0.21 log CFU/ml decline in bacterial population, respectively, (*P* ≤ 0.05). All bacterial strains except O157:H7 amp C declined in population to below the limit of detection after exposure to 5% v/v lactic acid for 300 s (Figure 4).

## Discussion

The overuse of antimicrobials in the food chain promotes the development of antimicrobial resistant bacterial strains (Bennani et al., 2020). In cattle production, antibiotics are used to treat diseases and promote growth in animals but long term exposure of bacteria to sub-lethal concentrations of antibiotics could exert selective pressure, favoring isolates with resistance genes or inducing cross-resistance to unrelated antibiotics (Capita et al., 2014; Oniciuc et al., 2019). In this study, resistance to ampicillin and a combination of ampicillin and streptomycin was induced in O157:H7 and non O157 STEC exposed to sublethal concentrations of each antibiotic over time. The resistance formats to these particular antibiotics were chosen based on previous studies with *E. coli* O157:H1730 where strains amp C and amp P Strep C had higher tolerance to lactic acid than the parent strain and other combinations of resistance to ampicillin, streptomycin and the combination of ampicillin and streptomycin using both chromosomal adaptation and plasmid-based transformation (Hayman et al., 2022; Oguadinma et al., 2022a,b). Plasmid based tolerance to ampicillin can increase tolerance to stressors in both *Salmonella* and STEC (Oguadinma et al., 2022b) and could have contributed to the increased tolerance to lactic acid that was observed in O157:H7 H1730 with amp P and strep C resistance.

The MIC of lactic acid was the same for both the O157:H7 and non-O157:H7 STEC evaluated indicating that the presence of an ABR profile does not impact resistance to lactic acid in STEC. Tolerance, which is defined as the ability of bacteria to sustain increased duration of exposure to an antimicrobial (Brauner et al., 2016) might be impacted. In a previous study, the presence of resistance to ampicillin and streptomycin in O157:H7 H1730 affected cross-tolerance to lactic acid (Oguadinma et al., 2022b). In this study, improved tolerance of O157:H7 H7130 with ABR profiles was also observed. Different bacterial serogroups might, however, vary in response to ABR associated cross-tolerance. ABR in the other strain variants evaluated did not significantly improve or diminish observed tolerance to lactic acid.

Tolerance to lactic acid due to ABR may be attributed to the activity of efflux pumps. In a previous study we observed that *E. coli* O157:H7 H1730 with multidrug resistance to ampicillin and streptomycin was more tolerant to lactic acid than the non ABR strain variant. The addition of the efflux pump inhibitor carbonyl cyanide m-chlorophenylhydrazone (CCCP) resulted in increased sensitivity to lactic acid (Oguadinma et al., 2022a). Efflux pumps modulate the activity of a large number of antibiotics and are transport proteins involved in the extrusion of toxic substrates from the internal to the external environment of cells (Van Bambeke et al., 2000; Webber, 2003; Swick et al., 2011). Efflux pumps have a broad substrate range and bacteria with antibiotic resistance over express these pumps (Webber, 2003). Expression of efflux pumps could also potentially vary between serogroups of *E. coli* and requires further exploration. In this study, all three variants of *E. coli* O121 showed comparable levels of lactic acid tolerance by bactericidal concentration to O157:H7 H1730 amp C and O157:H7 H1730 amp P strep C.

Cells that develop ABR were reported to spend an extended time in the lag phase (Fridman et al., 2014). Results from this study indicate that the impact of ABR on lag phase duration might be serogroup dependent as an extension in the lag phase duration due to ABR was only observed in strains O157:H7 H1730, O121:H19 and O26:H11. In the presence of lactic acid at sub-MIC, lag phase was significantly extended when bacterial strains were exposed to concentrations greater than 0.0625% v/v lactic acid for all the bacterial strains except O157:H7 H1730 with multidrug resistance (Figure 2A). The longest lag phase durations for all the bacterial strains were observed when the cells were exposed to 0.25% v/v lactic acid.

Previous studies have reported variations in acid resistance between STEC serogroups. Among the serogroups evaluated in this study, previous studies have reported O121 to be the most acid resistant (Large et al., 2005; Kim et al., 2015). In this study, all strain variants of O121 had bactericidal concentrations of 5% v/v lactic acid which was higher than the bactericidal concentrations observed for the other strains but comparable to the highly acid tolerant O157:H7 H1730 amp C and O157:H7 H1730 amp P strep C.

## Conclusion

The highest tolerance to lactic acid was observed in strains O121 NR, O121 amp C, O121 amp P strep C, O157:H7 H1730 amp C and O157:H7 H1730 amp P strep C confirming the high acid tolerance of O121 but also indicating that ABR in some STEC strains could improve acid tolerance.

The results highlight the impact of antibiotic resistance on growth parameters in STEC and the risk of antibiotic associated tolerance to sanitizers.

## Data availability statement

The raw data supporting the conclusions of this article will be made available by the authors, without undue reservation.

## Author contributions

IO performed the experiments and drafted the manuscript. AM conducted analysis of growth parameters and edited the manuscript. GD conceptualized the study, performed the experiments, and drafted and edited the manuscript. All authors contributed to the article and approved the submitted version.
